# The case for well-conducted experiments to validate statistical protocols for 2D gels: different pre-processing = different lists of significant proteins

**DOI:** 10.1186/1472-6750-5-7

**Published:** 2005-02-11

**Authors:** Sreelatha Meleth, Jessy Deshane, Helen Kim

**Affiliations:** 1 Biostatistics and Bioinformatics Unit, Comprehensive Cancer Center, University of Alabama at Birmingham, Birmingham, AL 35294, USA; 2 Department of Pharmacology, University of Alabama at Birmingham, Birmingham, AL 35924 USA; 3 Department of Pharmacology, University of Alabama at Birmingham, Birmingham, AL 35294, USA

## Abstract

**Background:**

The proteomics literature has seen a proliferation of publications that seek to apply the rapidly improving technology of 2D gels to study various biological systems. However, there is a dearth of systematic studies that have investigated appropriate statistical approaches to analyse the data from these experiments.

**Results:**

Comparison of the effects of statistical pre-processing on the results of two sample t-tests suggests that the results of 2D gel experiments and by extension the conclusions derived from these experiments are not independent of the statistical protocol used.

**Conclusions:**

This study suggests that there is a need for well-conducted validation studies to establish optimal statistical techniques to be used on such data sets.

## Background

The effort to produce an index of all human proteins (the human protein index, or HPI) began over twenty years ago. This project pre-dates the human genome project by more than a decade. However, the complexity of the task of creating this index was underestimated and the relative simplicity of the human genome with four known nucleic acids arranged in a linear coding order allowed the process of the sequencing of the human genome to progress exponentially [1]. The successful completion of the human genome project is now putting the focus back on proteins. The emergence of new and improved protein technologies from re-engineered two-dimensional (2D) gel systems to mass spectrometry has made the mapping and identification of the entire proteome of a cell (tissues) a much more accessible goal. Over the past few years a number of databases documenting the protein content of a single organism, organ or organelle have been created [2-6], and a number of papers describing results of experiments using these new and improved techniques have been published.

### The advantages of 2D gel technology

Two-dimensional electrophoresis is an extremely powerful tool for the analysis of complex protein mixtures. Proteins carry both positive and negative charges. The pH of the medium they are in determines their net charge. The pH that gives a zero net charge is the isoelectric point of the protein (pI). In Isoelectric Focusing (IEF), protein mixtures are electrophoresed in a gel containing a pH gradient. The proteins in the mixture migrate according to charge density until they reach the part of the gel that corresponds to their pI. At this point, their net charge is zero, and migration stops. This is the first dimension of separation in a 2D gel experiment. The electrophoresed gel is then layered on top of a polyacrylamide gel and electrophoresed once again. The proteins now move from top to bottom depending on molecular weight. The distance covered by a protein is inversely proportional to its size. This is the second dimension in a 2D gel. 2D is an effective method for identifying qualitative and quantitative differences between proteins expressed in various tissues or between tissues exposed to different experimental treatments. Although the number of proteins displayed by 2D is much lower than the estimated number of genes in a particular tissue, 2D is currently the only available technique that enables the isolation and separation of thousands of the individual proteins that constitute a tissue proteome [7]. Anderson et al [1] point out that although writing obituaries for 2D gels has become a popular past time, the supply of unrelated parameters applicable to protein separation is limited and nearly all other combinations have been explored in the past.

### Database for statistical analysis

Images of 2D gels are acquired into a database using an image scanner. Image analysis software converts the gel image into a digitised image in a computer, matches gels and spots on gels across the different groups and creates a database with information about spot intensity and spot location. As mentioned above, the two variables – the pI representing net charge of the protein and the molecular weight of the protein – are not correlated. In geometric terms this suggests that the two dimensions are orthogonal to each other. The two dimensions in a two-dimensional gel thus can be thought of as the two axes in a two dimensional graph. The coordinate on the x-axis is a measure of the isoelectric point (pI) of the protein, and the coordinate on the y-axis is a measure of the molecular weight of the protein

The information in the database includes a gel identification variable, a spot identification variable, the x and y coordinates of a protein spot and its intensity measured by the amount of light transmitted by the spot. Depending on the software package, one can obtain other parameters in the database, including a measure of the quality of the spot to various measures associated with spot intensity, such as volume, area, peak height, etc.

### The rationale for this study

The intensity of a protein spot is assumed to be directly related to the amount of protein in the particular tissue under investigation at that given time point. Changes in protein intensity are therefore approximated by changes in the intensities of protein spots in gel images. Changes in protein structure associated with post-translational modifications such as phosphorylation, oxidative modification or glycosylations may result in changes in the pI or molecular weight of the protein and are manifested in the gel by a change in the vertical or horizontal position. The object of 2D gel experiments is to detect differences in protein intensity/complexity between two groups of gels.

A number of recent publications [8-10] have used statistical models generally known as classifiers to detect differences in protein intensity/complexity between two groups of gels. Classifiers are increasingly being used in the analysis of hi-dimensional data sets derived from gene and protein expression experiments. These models help one to determine if the changes in protein intensity /complexity are specific enough to enable a clear separation of the gels into the right groups. They can also be used to provide a good visual demonstration of the differences between groups.

### Classifiers

Classification is the process of assigning objects to a category. An interest in classification permeates many scientific studies [11]. There are two broad categories of classification problems. In the first, e.g. discriminant analysis, one has data from known groups. Information that distinguishes these groups (i.e. differences in protein intensity/complexity) collected from an experiment is used to assign samples (gels) to these known groups. In the second case, e.g., cluster analysis, one has the information but no preset classification. The data is mined to see if there are naturally occurring clusters. These clusters are then investigated to identify commonalities within and differences between clusters. Stein and Zvelebil (12) and Patel et al (13) describe using 2D gel data sets to build supervised and unsupervised classifiers

Both types of classification problems have three stages, input, algorithm and output. Most published literature concentrates on the second of these. However, careful thought about what variables to use and how to characterize or summarize them as inputs into an algorithm are very important issues [11]. It is evident that the reliability and reproducibility of a classification is a function of the input, which in turn depends upon the process of data normalization, data reduction, and variable selection, i.e. the pre-processing of data. This paper focuses on the effects of preprocessing on the selection of variables that enters a classifier.

### Pre-processing

In order to conduct a systematic analysis of 2D gel data, one has to pre-process the data set. Pre-processing in the case of 2D gel analysis includes: 1) normalizing intensities to remove effects of differential loading and staining; 2) transformation of outcome variables to normally distributed variables; and 3) imputing values for missing spot intensities. We are not aware of any other study that has looked systematically at the effect of pre-processing 2D gel data on the results of subsequent statistical analysis of the data. In this paper, we present a protocol for the analysis of 2D gel data and examine the effect of statistical pre-processing of 2D gel datasets.

**Figure 1:**
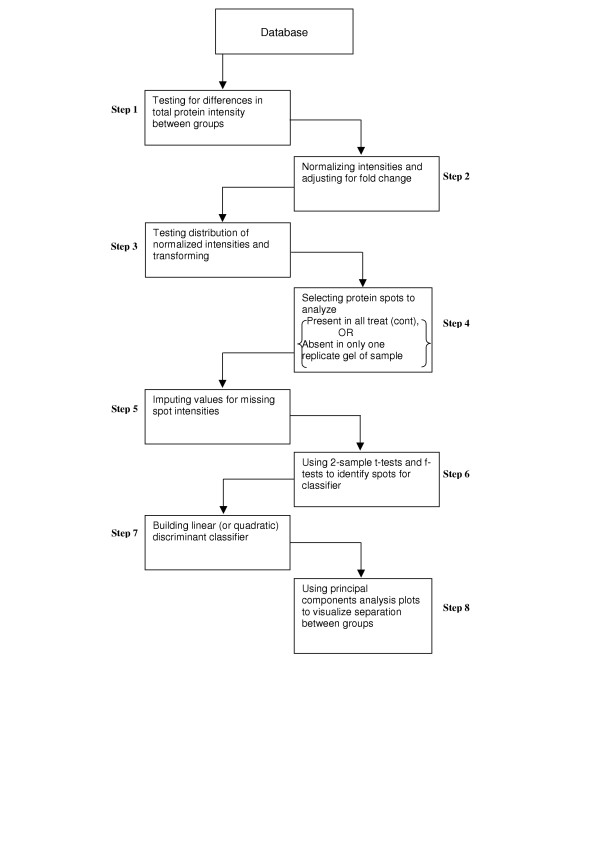
**Statistical protocol for 2D gels. **The first protocol that we followed in the statistical analysis of data from 2D gel experiments is demonstrated in the flowchart in Figure 1. This consists of: 1) testing for differences between the groups with respect to total protein expression; 2) normalizing protein intensities on a gel to the mean total intensity of its group (e.g. treatment or control); 3) expressing each normalized intensity as a fraction of the total protein intensity in the experiment in order to make fold change comparisons meaningful; 4) testing the distribution of normalized intensities and using appropriate transformations if necessary to convert distribution to a normal distribution; 5) selecting the subset of protein spots to analyse; 6) imputing values for missing spot intensities; 7) using 2-sample t-tests and f-tests to identify protein spots that can be used to build classifiers; 8) building a linear (or quadratic) discriminant classifier; and 9) using Principal Components Analysis plots to demonstrate the separation between groups visually.

The effect of using two different formulas for normalizing versus not normalizing, log-transformation versus no log-transformation and single value imputation versus multiple value imputation, and averaging spot intensities across replicates versus keeping the replicate information separate are compared using the results of two sample t-tests. We also compare the results of two-sample t-tests provided by the image analysis software PDQUEST to results obtained after following the protocol described in Figure 1. PDQUEST allows the user to normalize the data but has no facility for testing the distribution of the outcome variable and transforming it to fit a normal distribution. To the best of our knowledge, PDQUEST replaces missing spot intensities with a zero.

### The experiment

The data used in this study is from an experiment that looked at the effect of a diet enhanced with grape seed extract on the proteome of whole brain homogenates of Sprague-Dawley rats [10]. There were five treated animals and five control animals. Due to sample availability and other issues related to the creation of 2D gels, each biological replicate had different numbers of technical replicates. The maximum number of replicates was four, and the minimum was two. A number of changes in proteins that were attributed to treatment differences in this study have been identified with Matrix Assisted Laser Desorption Ionization – Time Of Flight (MALDI-TOF) Mass Spectrometry. These changes have also been confirmed in later experiments with transgenic mice. Thus the protein changes detected by the statistical protocol used in this study have been shown to be biologically valid and relevant to the systems being studied.

## Results

### Variability in the resolution of protein spots in 2D gels

The resolution of protein spots in a 2D gel is highly variable. It can differ considerably between technical replicates of the same biological sample. Samples 6, 7, 8, 9, and 10 were the five biological replicates in the treatment group. Samples 22, 23, 24, 25, and 26 were the five biological replicates in the control group. Table 1 demonstrates the breakdown of the resolved protein spots in the different samples and its replicates. Biological sample 7, for instance, had 546 protein spots resolved in at least one of its four technical replicates. 169 (31%) proteins occurred in all the four replicates. An additional 130 (26%) were present in at least three replicates out of four. A further 97(18%) were present only in two replicates out of four, and 150 (27%) were present only in one of the four replicates.

### Low correlation between technical replicates

Table 2 displays the range of Pearson's correlation coefficients and Kappa coefficients between the technical replicates of the same sample. The correlation coefficient here is a measure of the association between the spot intensities on technical replicates. The correlation between technical replicates is not very high. The correlation coefficients range from a high of 0.93 to a low of 0.47. The r-square ranges from 87% to 22%. The Kappa coefficient measures the degree of agreement between the spots present on two replicate gels of the same sample. A zero indicates no agreement and one indicates perfect agreement. If the confidence interval spans zero then the hypothesis that there is no agreement between the replicate gels cannot be rejected. Ten of the sixteen separate confidence intervals in Columns 5 and 6 of Table 2 include zero. This suggests no agreement between the replicate gels of most samples.

#### Assessing the quality of a pre-processing technique

In Tables 3 to 10 the spot identification numbers in bold represent proteins that were subsequently identified and found to be biologically relevant to the system being studied (10). In all, eleven proteins that had significantly different intensities at alpha = 0.05 were identified. The measure of the quality of a particular pre-processing technique in this study was the proportion of these eleven proteins identified as statistically significant in a two-sample t-test after the particular technique was used.

### The effect of log-transformation and minimum values substitution on the distribution of intensities

#### Log – Transformation

Figures 3a and 3b are the QQ plots for the raw spot intensity and normalized spot intensities for the 201 protein spots on a representative gel from the control group. These plots demonstrate that the normalization technique used does not alter the basic distribution of the raw data. They also demonstrate the highly non-gaussian distribution of the spot intensities. Figure 3c demonstrates that the log transformation converts the distribution of the intensities from a very non-normal distribution to a normal distribution. The points lie very close to the straight line that represents a normal distribution. We have found that the log transformation reduces the skew in the distribution of the spot intensities if the image analysis is done in PDQUEST^® ^and most data sets produced by the software PROGENESIS^®^. Only one out of seven 2D gel data sets analysed by us so far did not respond well to this transformation. A closer examination of this data showed it had a large number of saturated spots, and thus needed to be rerun. The transformation of the distribution of the 201 spots also worked reasonably well at the level of individual spot intensities. This was important to confirm since the two sample tests were done at individual spot level. In cases where there was a considerable skew in the distribution, e.g., SSP 1733, the log transformation made the distribution of spot intensities normal. The Anderson-Darling test for normality for SSP 1733 in the control group, has a p-value = 0.03 before the transformation, and p-value = 0.456 after the transformation. In general, this was true of most spots we examined.

Table 3 compares the t-test results of non-log-transformed normalized spot intensities where the missing spot intensities were replaced with a zero; to the results using normalized spot intensities that were log-transformed. In the non-log transformed data without normalization, six of the eleven spots known to be significantly different were picked up. Six of the eleven proteins were picked after log transformation of the non-normalized data as well. It is important to note that the two lists of six proteins were not identical. After normalization, in the log-transformed data the t-test picks up all the eleven proteins, whereas, in the non-log-transformed data seven proteins are picked up (Table 4).

#### Effect of normalization

Table 5 compares the proteins that were found to be significant (p = 0.05) in a t-test when the spot intensities were not normalized, not log transformed, and the missing intensities were replaced with a zero (Column 1); proteins that were found to be significant in a t-test (p = 0.05) when the spot intensities were normalized using normalization 1 (see methods), not log transformed, and the missing intensities were replaced with a zero (Column 2); proteins that were found to be significant in a t-test (p = 0.05) when the spot intensities were normalized using normalization 2 (see Methods), not log transformed, and the missing intensities were replaced with a zero (Column 3); and the proteins that were found to have significantly different intensities by the image analysis software PDQUEST (Column 3), which normalizes (each intensity divided by the total intensity of the gel) the data but does not use a log transformation. As is evident from comparisons of Column 1 with Columns 2 and 3 in Table 1, normalization has an effect on the number of proteins that are detected. The difference in Columns 2, 3 and 4 suggests that the protocol used for normalizations also has an impact on the proteins that are picked up as significant. In non-log transformed data, data sets with no normalization, normalization 1 and normalization 2 picked up six of the eleven proteins, and PDQUEST method picked up seven of the eleven proteins. Over fifty percent of the proteins picked up by PDQUEST occurred in very small numbers of gels, and hence did not meet our selection criteria for inclusion into the analysis data set. A number of the other proteins picked up by PDQUEST had skewed distribution. After log-transformations these proteins were no longer statistically significantly different. Tables 6, 7 and 8 again demonstrate that normalization 2 has a significant impact on the number of proteins identified as significantly different in intensity. In all of these tables, it is important to note once again that the highlighted proteins in each column vary with the pre-processing technique.

#### Effect of imputation of missing spots

In Table 9, Columns 1, 2, and 3 offer a comparison of the spots identified as significantly different (alpha = 0.05) when the three different kinds of imputation of missing spots were used. The three different kinds of imputation did not make much difference to the spots identified as significantly different in intensities at alpha = 0.05. In fact in this case we have identical lists in all three columns of table 9. The purpose of using multiple imputation instead of single value imputation, however, has more to do with getting a better estimate of the variance of a quantity than with the correct estimation of the mean. Since the t-statistic is a function of both the difference in means as well as the variance of a variable, given a constant mean, underestimating variance would lead to false positives, and overestimating variance would lead to false negatives. This data set shows a good example of the degree of variability on the intensities. Table 10 demonstrates that single value imputations tend to either underestimate (var = 0 for SSP 6452), or over-inflate the estimates of variance (e.g. SSP 1509). The variance of SSP 1733, which has no missing spot intensities, gives us a rough idea of the degree of variability expected in spot intensities when there are no intensities missing. The estimates of variance for the proteins with missing intensities are much closer to the values seen in SSP 1733 when one uses a multiple imputation technique.

#### Averaging across replicates versus keeping replicates separate

Given the lack of association between technical replicates, we used replicate information in two ways: 1) Spot intensities were averaged across the replicates so that the t-tests compared average spot intensities in the five treatment samples versus five control samples, and 2) The replicate gels of each sample were treated as independent gels, and the t-tests compared spot intensities in the fifteen treatment gels to the intensities in the fifteen control gels. Columns 1 and 2 in Table 11 compare Method 1 above to Method 2. Six out of eleven proteins are picked up using Method 1, whereas all ten are identified as significantly different if the replicates are kept separate.

## Discussion

### Normalization

Differential sample loading and stain absorption and other process variables can contribute to variability in measured protein intensity. In order to ensure that the detected differences in protein intensity are not due to a "technical" variability introduced by the process of gel creation, spot intensities are "normalized." Dividing the intensity of each protein on a gel by the total protein intensity of that gel is a widely used technique to reduce the "individual gel effect" on protein intensities [14]. Normalizing the data is an important step in many datasets, but it becomes especially important in proteomics experiments, which in general have many more variables than samples. In this case a systemic error in processing samples or gels that affects only one or two gels can have a huge impact on the results. This study has demonstrated that the results of statistical tests are not independent of the normalization technique.

### Testing for normality and transforming data

To the best of our knowledge, none of the image analysis software packages available to date provide the tools necessary test for the distribution of the data. All of them provide t-tests or the non-parametric Wilcoxon rank sum test or ANOVA to test for differences in individual spot intensities. The probability values (p-values) for differences between groups are based on the assumptions of the normality of the distribution of spot intensities and equal variances. In order to make an informed judgement about the validity of the p-values of the tests above, it is important to know if these assumptions are met [15,16]. If one uses the averaged spot intensities across gels, the argument could be made that the central limit theorem obviates the need for a log transformation. Given the highly skewed nature of the raw spot intensities, the dependence of the mean and the variance of intensities, and the fact that even the non-parametric Wilcoxon test assumes symmetry in the outcome variable [17], log transformation of the data is still advisable.

### Missing spot intensities

Despite the fact that 2D-gel technology offers many advantages, one of the pitfalls associated with this technology is the need for several replicates for proper validation of results. There are instances where one does not observe reproducible spot patterns or individual proteins even in replicate gels of the same sample. Missing spot intensities are commonly observed in 2D gel datasets. Multivariate techniques such as Principal Component Analysis and Discriminant Analysis (DA) are ideal tools to use on databases that have multiple outcome variables (protein intensities). However, SAS or any other statistical software that is used to analyse the data using multivariate techniques such as PCA and DA requires data sets with non-missing values. Gels with missing spot information will thus be dropped from the analysis. Since all gels will have some spot information missing, this will result in no gels being available for data analysis. To the best of our knowledge, all image analysis software packages substitute zeroes for missing intensity values. Missing intensities may be caused by the fact that a protein spot truly does not exist in one group compared to the other or because the spot intensity is so low that it is not detected by the image analysis software. In this study we treated all missing spots as undetectable spots. The question we were trying to answer was "If a spot exists but is 'undetectable', what is the best 'detectable' value to substitute as its intensity?" The most intuitive value was the smallest "detectable" intensity in the experiment hence we used the lowest intensity value in the experiment for the single value imputation. However, substituting a single value for all missing spot intensities would skew the distribution of spot intensities considerably. Thus the second option was to use a random process to substitute missing spot intensities with a plausible set of "detectable" intensities. We created a set of lowest 'detectable' intensities from the lowest intensity on each of the thirty gels. These values were used to impute missing intensity values as described in the methods section. Although we have used the terms "imputation" and "multiple imputation", these terms are not to be equated with multiple imputation advocated by Rubin [18]. Rubin's techniques assume the missing intensities to be Missing at Random (MAR). By our assumption, the spots are undetectable because of the intensity level, or the probability of missing is a function of the intensity. This by Rubin's definition would make the missing spot intensities Non-Ignorable Non-Response. His multiple imputation techniques thus would not be valid in this context.

The issue of missing protein intensities is one that has not been addressed at all in the literature describing 2D gel studies. In this study we treated all missing intensities as the same. However, all missing intensities in 2D gels are not equal. Some missing intensities are missing because they truly do not exist in one group versus the other, whereas others are missing because of the inherent variability in the process of creating 2D gels. This suggests that one needs to approach the filling of missing values differently based on the probability of a spot being a truly missing protein or one that is missing due to the process of gel creation. One way to do this would be to assume that spots that occur in two out of three replicates (or three out of four replicates) of a sample are true spots. These missing intensities would then be replaced by the mean value of the remaining two (three) spots. On the other hand, the assumption that those proteins that are missing in all control gels or all treatment gels are proteins that are turned on or off is justifiable. This in turn suggests that for this set of proteins the random imputation of missing values with a set of plausible minimum intensity values, acting as placeholders so that the non-missing data can be used in analyses, can also be justified.

#### Which method should one use?

In the last few years, published reports of 2D gel analysis have concluded that heart failure was associated with protein modifications in three cellular systems [19], identified proteins expressed in six different regions of brains of Alzheimer's disease patients [7], had been used to establish genetic relationships in the Brasscacae family [20], to classify human ovarian tumours as malignant and benign [8], in the detection of polypeptides associated with the histo-pathological differentiation of primary lung cancer [21], and to identify eight protein feature changes that differentiated breast cancer cell lines that did or did not form tumours in nude mice [9]. These are all important studies that will be used as springboards to launch ever more expensive and sophisticated experiments. We have demonstrated that protein changes that are large (e.g. SSP 6452 present in controls and absent in all treatment gels) are independent of the statistical protocol used. The identification of more subtle changes can vary widely depending on the statistical algorithm used to pre-process and analyze the data. Our experience with the GSE data and a couple of subsequent experiments we have been involved in suggests that the algorithm we have developed is more sensitive with respect to identifying biologically relevant proteins that image analysis software might miss. However, it is a fact that pre-processing could also give rise to false positive results. It is important to establish the best statistical protocol for analysing the data from these studies. One way to get around the issue of the effect of pre-processing is to restrict a study to only those proteins that are picked up as significant by image analysis software. As we have mentioned above, in this study a large number of the spots selected by PDQUEST were poor quality spots either in terms of protein quality or consistency. Another option is to consider only those spots that appear in two sets of analyses (e.g. image analyses, and the protocol described here) as true changes. One is thus restricting oneself to gross changes. As the use of proteomic techniques moves forward, however, we think it will be important to identify more subtle changes in proteins. A number of studies have suggested that change in protein expression that starts the cascade of changes that leads to a diseased tissue need not always be gross or dramatic. Subtle changes in expression early in a pathway can cause significant changes downstream. Shapiro et al [22] suggest that subtle changes in the spatial or temporal expression of the patterning molecule Sonic Hedgehog (SHH) is linked to the proliferation and patterning of developing limbs. Similarly, a disease condition could be caused by small changes in expression in a number of proteins. Reneiri et al [23] suggest that the phenotypic expression of Retts in some but not all girls with the MECP2 mutation suggests that MECP2 causes deregulation of a very small subset of genes that have not yet been detected or that very subtle changes in many genes (by extension proteins) may cause the neuronal phenotype. The importance of picking up subtle changes in protein expression suggested by studies cited above point to a need to establish a way to identify the optimal statistical pre-processing techniques for 2D gel datasets.

An intuitively appealing way to do this is to create 2D gels with serially diluted quantities of commercially available proteins, establishing the relationship of protein quantity to spot intensity and then proceeding to compare different statistical pre-processing techniques to the datasets acquired from these gels. Since it is a known fact that commercially bought proteins may not necessarily be pure and may be present in multiple modified forms due to the process of isolation, the experiment described above may be enhanced by using controlled biological samples from a cell culture with at least a hundred resolved spots. Once again, the sample could be loaded in on gels in known concentrations and the process described above would be repeated. Clearly, the set of pre-processing techniques that picks up differences that come closest to the true differences would be chosen as the optimal techniques. Some recent publications have used similar techniques to establish the validity of emerging proteomic technology. Alban et al [24] used an *Escherichia coli *lysate "spiked" with varying amounts of four different known proteins to test a novel experimental design that exploits the sample multiplexing capabilities of DIGE by including a standard sample in each gel. Rabilloud et al [25] compared the staining sensitivity of RuBPS and Sypro Ruby of serial dilutions of molecular weight markers. However, there are no designed experimental studies that have looked at the impact of statistical pre-processing or the effectiveness of various statistical techniques on the conclusions drawn from 2D gel experiments. Given the proliferation and promise of 2D gel experiments, we suggest that the need to conduct these validation experiments is urgent.

In this study we have used the proteins that were subsequently identified by MALDI-TOF spectroscopy as a measure of how well particular statistical protocols perform. We concede that there is an inherent bias in that the spots that were identified by MALDI-TOF were selected on the basis of the protocol described in this paper. The described protocol therefore will seem to perform much better than others in this comparison. This however does not diminish the main thrust of this paper, which is that statistical protocols affect the conclusions drawn from a 2D gel experiment.

## Conclusions

**Figure 2:**
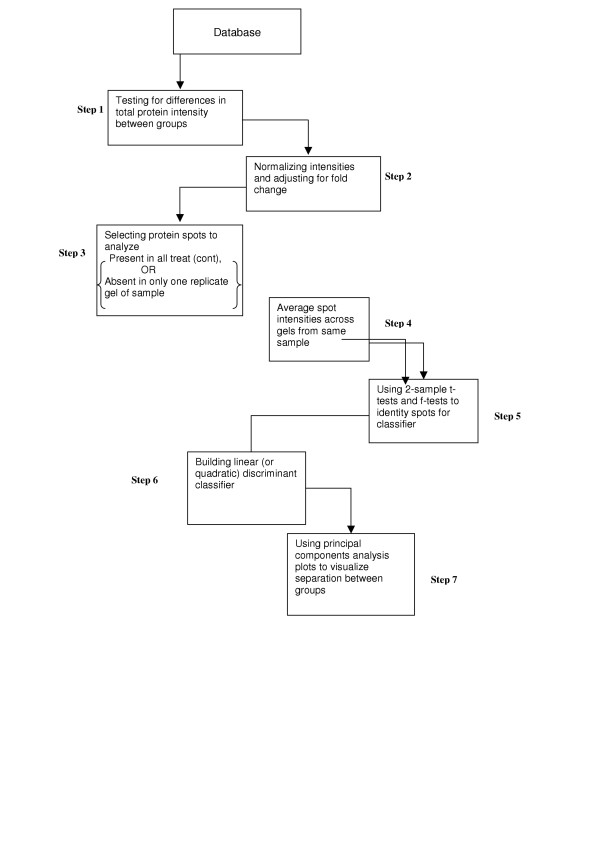
**Statistical protocol for 2D gels. **The second protocol that we followed in the statistical analysis of data from 2D gel experiments is demonstrated in the flowchart in Figure 2. This consists of: 1) testing for differences between the groups with respect to total protein expression; 2) normalizing protein intensities on a gel to the mean total intensity of its group (e.g. treatment or control); expressing each normalized intensity as a fraction of the total protein intensity in the experiment in order to make fold change comparisons meaningful; 3) selecting the subset of protein spots to analyse; 4) Average spot intensities across gels from the same sample; 5) imputing values for missing spot intensities; 6) using 2-sample t-tests and f-tests to identify protein spots that can be used to build classifiers; 7) building a linear (or quadratic) discriminant classifier; and 8) using Principal Components Analysis plots to demonstrate the separation between groups visually.

**Figure 3:**
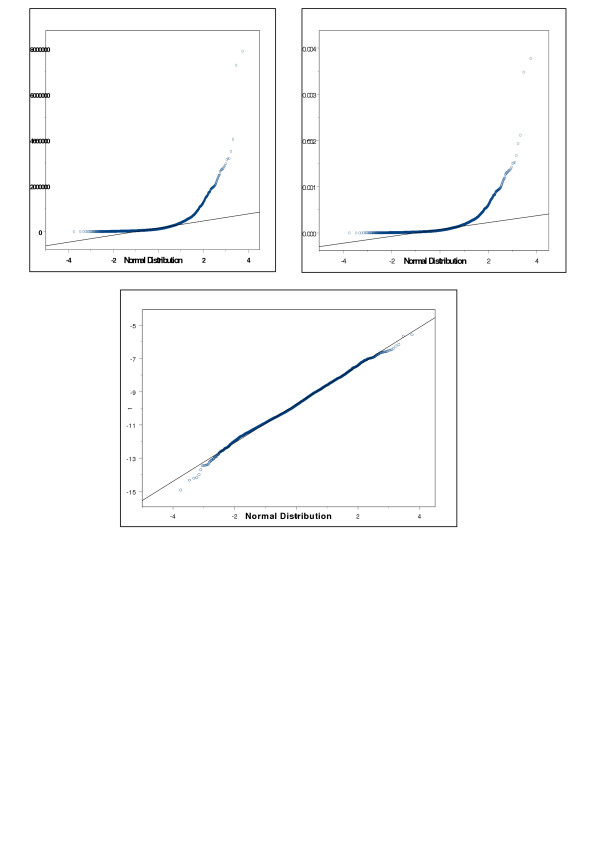
**Intensity plots. **(a) Plot of raw intensities before log-transformation and normalization. Probability plot of raw intensities of 201 spots in the final data set; a normally distributed variable is expected to plot a line close to the straight line; the intensities are very skewed. (b) Plot of Normalized spot intensities. Probability plot of normalized spot intensities of 201 spots in the final data set. Comparison of 3a and 3b demonstrates that the normalization does not alter the distribution of the spot intensities. (c) QQ plot of log transformed intensities. Figure 3c demonstrates that the log transformation successfully transforms the highly skewed distribution of spots into a normal distribution.

This study has demonstrated that the pre-processing of the data from 2D gel experiments can have a significant impact on the results of statistical tests. The purpose of the study was not to identify the particular statistical protocol used in this study as the optimal protocol, but rather to demonstrate that the results and conclusions from a biological experiment are not independent of the statistical protocol. The study has in effect looked at three different statistical protocols. The one described in Figure 1, the one described in Figure 2, in which the averaging of intensities across replicates allows one to proceed directly on to the t-tests, without the steps of testing distributions, or imputation of missing spots, and the protocol used by PDQUEST. Allowing for the inherent bias we have described above, this study shows that the protocol described in Figure 1, with normalization technique 2, and multiple imputations, is superior to the method used by PDQUEST, or the one in Figure 2. Given the possible bias in this study, the larger conclusion from the study is that there is a great need for research into developing optimal statistical methodology to analyze data from 2D gel experiments.

## Methods

We used data from an experiment that compared the protein expression in the whole brain homogenate of rats that were fed a diet with 5% of grape seed extract to that of a normal rat diet. This experiment will be described in a separate article. The data from this experiment was exported to a database, which was saved as a text file. The database was imported into SAS V-9.0 (Statistical Analysis Software, 2003 Cary NC, USA), which was then used to do all the statistical analysis.

### Normalization 1

The intensity of each protein spot was divided by the total protein content (total intensity) in the experiment.

### Normalization 2

The intensity of each spot in each group was then normalized to the median intensity of its group, i.e. the intensity of each spot was converted into the intensity it would have had if the gel it was in had a total intensity equal to the median total intensity of the group. This is described by the following formula:


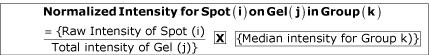


This normalization reduces intra group variability, but maintains the inter group variability.

Fold change is a common metric used in articles describing gene array and proteomic experiments. Fold change measures the degree of change in protein intensity in the treatment group, compared to the control group. This is measured by dividing the average spot intensity in the treatment group by the average spot intensity in the control group. In order for this ratio to be a true comparison of the intensities in the two groups, the intensities need to be expressed as proportions of the same quantity, i.e. they need to be divided by the same quantity. Normalization 1 has this property built into the formula. In normalization technique 2, the normalized intensities were divided by the total protein content in the experiment in order to make a fold change comparison meaningful.

### Subset of spots included in analysis

A spot was considered present only if it was present in both replicates of a sample if there were only two replicates, in two out of three replicates if there were three replicates of a sample, and three out of four replicates if there were four replicates of a sample. We also included spots that were present in all controls and absent from all treatments, or present in all treatment gels but absent from all controls. Using these criteria, we had 201 spots that were available for statistical analysis from the GSE database.

### Missing spot intensities

Given the criteria used to subset spots for the final analysis, there was a large number of missing spot intensities. We examined the effect of filling in the missing spots in three different ways.

1. Missing spot intensities were first replaced with the lowest value of log-transformed intensities. In this case the value was -17.28. The effect of the replacement of this single value for all missing spot intensities is the same as the effect of replacing non-log transformed spot intensities with zero.

2. We created 1000 separate data sets that randomly selected one of the 15 lowest spot intensity values from the 15 gels in the control group to replace each of the missing spot intensities in the controls, and repeated the process for the 15 treatment gels.

3. In the third imputation method, each missing spot intensity was replaced with a randomly selected value from the 30 lowest values from each gel in the experiment without regard to whether the value chosen was from a control or treatment gel. The two methods of random imputation of spot intensities were replicated 1000 times. two sample t-tests were repeated with each replication.

## Authors' contributions

HK is the PI of the project that provided the data for this experiment. JD, HK's Research Associate, created the 2D gels, conducted the image analysis and created the database. SM designed this study and performed the statistical analyses.

## Figures and Tables

**Table 1:** The number of protein spots resolved in replicate gels of the same biological sample.

**Sample No (Treatment group)**	**No. of Replicates**	**Total Proteins resolved (occurring in at least one replicate) (%)**	**Number resolved in all replicates (%)**	**Number resolved in all but one replicate (%)**	**Number resolved in all but two (%)**	**Number resolved in just one (%)**
**6 (GSE)**	3	4064 (100)	309 (8)	563 (14)	3192 (78)	-
**7 (GSE)**	4	546 (100)	169 (31)	130 (26)	97 (18)	150 (27)
**8 (GSE)**	4	954 (100)	186 (19)	120 (12)	105 (11)	543 (57)
**9 (GSE)**	2	904 (100)	342 (39)	562 (62)	-	-
**10 (GSE)**	2	396 (100)	229 (58)	167 (42)	-	-
**22 (CONT)**	4	924 (100)	234 (25)	89 (10)	109 (12)	492 (53)
**23 (CONT)**	4	950 (100)	161 (17)	151 (16)	102 (11)	536 (59)
**24 (CONT)**	2	879 (100)	312 (35)	567 (65)	-	-
**25 (CONT)**	3	957 (100)	272 (28)	117 (12)	568 (59)	
**26 (CONT)**	2	432 (100)	183 (42)	249 (58)	-	-

The variability in the resolution of protein spots in technical replicates in a 2D gel experiments is one of the causes of the large number of missing spot intensities. The variability in the table above demonstrates the need for technical replicates as a quality control measure to identify spots that are most 'reliable' and common and therefore most useful to generalize to a larger population.

**Table 2:** Highest and lowest correlation among spot intensities between technical replicates in a 2D gel experiment; highest and lowest Kappa coefficients between technical replicates

**Sample**	**No of Replicates**	**Highest Correlation Coefficient (R-sq%)**	**Lowest Correlation Coefficient (R-sq%)**	**Highest Kappa 95% CI**	**Lowest Kappa 95% CI**
6 (GSE)	3	0.774 (60%)	0.547 (30%)	0.4962 (-0.1039,1.000)	0.1476 (-0.1374,0.4326)
7 (GSE)	4	0.907 (82%)	0.589 (35%)	0.4437 (0.1004,0.7870)	-0.0767 (-0.114,-0.0350)
8 (GSE)	4	0.932 (87%)	0.617 (38%)	0.4445 (0.1027,0.7864)	0.0099 (-0.0855,0.1052)
9 (GSE)	2	0.747 (56%)	0.747 (56%)	0.1299 (-0.1384,0.3982)	0.1299 (-0.1384,0.3982)
10 (GSE)	2	0.837 (70%)	0.837 (70%)	0.1231 (-0.0486,0.2948)	0.1231 (-0.0486,0.2948)
22 (CONT)	4	0.805 (65%)	0.467 (22%)	0.6538 (0.3705, 0.9372)	0.2599 (-0.0566,0.5765)
23 (CONT)	4	0.845 (71%)	0.632 (40%)	0.3269 (0.0304,0.6235)	0.0322 (-0.0296,0.0941)
24 (CONT)	2	0.711 (50%)	0.711 (50%)	0.0743 (-0.1284,0.2770)	0.0743 (-0.1284,0.2770)
25 (CONT)	3	0.837 (70%)	0.524 (27%)	0.2843 (0.0073,0.5613)	0.2384 (-0.0629,0.5397)
26 (CONT)	2	0.578 (33%)	0.578 (33%)	0.1946 (0.0531,0.3361)	0.1946 (0.0531,0.3361)

The Pearson correlation coefficient is a measure of the linear relationship between two variables. R square, the square of Pearson's correlation is a measure of how much variability in one variable is explained by the variability in the other. Since technical replicates are expected to be identical, the r-squares are expected to be very high, at least 0.95. The table demonstrates the degree of variability between technical replicates after normalization. The Kappa coefficients with the 95% confidence intervals confirm the same thing. Ten out of sixteen confidence intervals span zero, indicating no agreement between technical replicates of the same sample in those cases.

**Table 3:** The effect of log transformation using non-normalized data.

**No Log Transform No Normalization, Missing replaced with zero (55% of identified spots picked up)**	**Log Transformed No Normalization, Missing replaced with – 17.28 (55% of identified spots picked up)**
**SSP 1509**	**SSP 1509**
**SSP 1733**	
	**SSP 2307**
	**SSP 3219**
	**SSP 3806**
**SSP 4225**	
**SSP 4435**	**SSP 4435**
SSP 4438	
SSP 4519	
SSP 4724	
**SSP 5413**	
SSP 6314	SSP 6314
**SSP 6452**	**SSP 6452**

Column 1 has spots that have significantly different intensities (p = 0.05) before normalizing and log transforming data. Column 2 has spots that are significantly different in intensity before normalizing data, but after using a log transformation. Spots in **bold **were later identified by MALDI-TOF. These were all spots that were biologically relevant to the system being studied. The percentages in parenthesis measure in the header indicate how many of the ten proteins known to be different were identified before log transformation.

**Table 4:** The effect of log transformation using non-normalized data.

**No Log Transform Normalized, Missing replaced with zero (63% of identified spots picked up)**	**Log Transformed Normalized, Missing replaced with – 17.28 (100% of identified spots picked up)**
	SSP 1134
**SSP 1509**	**SSP 1509**
	**SSP 1733**
**SSP 2309**	**SSP 2309**
	**SSP 3219**
	**SSP 3806**
	**SSP 4203**
**SSP 4225**	**SSP 4225**
**SSP 4435**	**SSP 4435**
SSP 4519	
	SSP 4724
SSP 5309	
SSP 5329	
**SSP 5413**	**SSP 5413**
**SSP 6205**	**SSP 6205**
SSP 6228	
SSP 6304	SSP 6304
SSP 6314	SSP 6314
SSP 6321	
SSP 6349	
SSP 6443	
**SSP 6452**	**SSP 6452**
SSP 7223	SSP 7223
SSP 7334	
SSP 7750	SSP 7750
SSP 8613	

Column 1 has spots that have significantly different intensities (p = 0.05) after normalizing and log transforming data. Column 2 has spots that are significantly different in intensity after normalizing data (using normalization 2), but after log transformation. The percentages in parenthesis in the header measure how many of the ten proteins known to be different were identified after log transformation and normalization.

**Table 5:** The effect of log transformation using non-normalized data.

**No Log Transform No Normalization Missing replaced with zero (54% of identified spots picked up)**	**No Log Transform – Normalization 1 Missing replaced with zero (54% of identified spots picked up)**	**No Log Transform Normalization 2, Missing replaced with zero (54% of identified spots picked up)**	**No Log Transform Normalized-PDQUEST (64% of identified spots picked up)**
			SSP 0312^1^
			SSP 1112^1^
			**SSP 1309**
			SSP 1321^1^
			SSP1331^1^
**SSP 1509**	**SSP 1509**	**SSP 1509**	
**SSP 1733**	**SSP 1733**		
			**SSP 2307**
		**SSP 2309**	**SSP 2309**
			SSP 3234^1^
			SSP 3437^1^
			SSP 3523^1^
**SSP 4225**	**SSP 4225**	**SSP 4225**	**SSP 4225**
**SSP 4435**	**SSP 4435**	**SSP 4435**	
SSP 4438	SSP 4438		SSP 4438^2^
			SSP 4517^1^
SSP 4519	SSP 4519	SSP 4519	SSP 4519^2^
			**SSP 4637**^2^
SSP 4724	SSP 4724		SSP 4724
			SSP 4735^1^
			SSP 5011^1^
		SSP 5309	
		SSP 5329	
**SSP 5413**	**SSP 5413**	**SSP 5413**	**SSP 5413**
		SSP 6205	
		SSP 6304	
SSP 6314		SSP 6314	SSP 6314
		SSP 6321	
		SSP 6349	
		SSP 6443	
**SSP 6452**	**SSP 6452**	**SSP 6452**	**SSP 6452**
		SSP 7027	
			SSP 7231
		SSP 7223	
		SSP 7334	
			SSP 7413^1^
		SSP 7750	
		SSP 8613	

^1 ^These are spots that were present in a very small number of gels, and therefore did not meet our criteria to be included.

^2 ^These spots have highly skewed distributions or were very poor quality spots. Log transformation made out the distribution closer to normal and p-values were no longer significant.

Column 1 has spots that have significantly different intensities (p = 0.05) normalizing and log transforming data. Column 2 has spots that are significantly different in intensity after using normalization 1, but before using a log transformation. Column 3 has spots that are significantly different in intensity after using normalization 2, but before using a log transformation. Column 4 has the results from the image analysis software PDQUEST, which has an option for normalizing but no log transformation. Columns 1 and 2 are subsets of the 201 spots in the final data set that met our criteria for inclusion. Column 3 is a subset of all possible spots in the experiment. Spots in **bold **were later identified by MALDI-TOF. These were all spots that were biologically relevant to the system being studied. The percentages in parenthesis in the header measure how many of the ten proteins known to be different were identified after the different normalization techniques.

**Table 6:** T-Test results of log transformed intensities pre-and post normalization – Imputation Method 1

**Normalization 1 Missing replaced by = -.17.28 Log transformed (36% of identified spots picked up)**	**Normalization 2 Missing replaced by = -17.28 Log transformed (100% of identified spots picked up)**
	SSP 1134
**SSP 1509**	**SSP 1509**
	**SSP 1733**
	**SSP 2309**
	**SSP 3219**
**SSP 3806**	**SSP 3806**
	**SSP 4203**
	**SSP 4225**
**SSP 4435**	**SSP 4435**
SSP 4724	SSP 4724
	**SSP 5413**
	**SSP 6205**
	SSP 6304
SSP 6314	SSP 6314
**SSP 6452**	**SSP 6452**
	SSP 7223
	SSP 7750

The table shows the results of two sample t-tests on log-transformed intensities, pre and post normalization, when all missing intensities were replaced by the lowest intensity value in the experiment (-17.28). Spots in **bold **were later identified by MALDI-TOF. These were all spots that were biologically relevant to the system being studied.

**Table 7:** T-Test results of log transformed intensities pre-and post normalization – Imputation Method 2

**Normalization 1 Missing replaced by Random sample of minimum from 15 gels in each group Log transformed (36% of identified spots picked up)**	**Normalization 2 Missing replaced by Random sample of minimum from 15 gels in each group Log transformed (91% of identified spots picked up)**
	SSP1134
**SSP 1509**	**SSP 1509**
	**SSP 1733**
	**SSP 2309**
**SSP 3806**	**SSP 3806**
	**SSP 4203**
	**SSP 4225**
**SSP 4435**	**SSP 4435**
SSP 4724	SSP 4724
	**SSP 5413**
	**SSP 6205**
	SSP 6228
	SSP 6304
SSP 6314	SSP 6314
**SSP 6452**	**SSP 6452**
	SSP 7223
	SSP 7334
	SSP 7750

The table shows the results of two sample t-tests on log-transformed intensities, pre and post normalization, when each missing intensities in GSE (control) gel were replaced by randomly selecting one of the 15 lowest spot intensity values from the 15 gels in the GSE (control) group. Spots in **bold **were later identified by MALDI-TOF. These were all spots that were biologically relevant to the system being studied.

**Table 8:** Test results of log transformed intensities pre-and post normalization – Imputation Method 2

**Normalization 1 Minimum replaced by Random sample of minimum from all 30 gels Log transformed (36% of identified spots picked up)**	**Normalization 2 Minimum replaced by Random sample of minimum from all 30 gels Log transformed (91% of identified spots picked up)**
	SSP 1134
**SSP 1509**	**SSP 1509**
	**SSP 1733**
	**SSP 2309**
**SSP 3806**	**SSP 3806**
	**SSP 4203**
	**SSP 4225**
**SSP 4435**	**SSP 4435**
SSP 4724	SSP 4724
	**SSP 5413**
	**SSP 6205**
	SSP 6228
	SSP 6304
SSP 6314	SSP 6314
**SSP 6452**	**SSP 6452**
	SSP 7223
	SSP 7334
	SSP 7750

The table shows the results of two sample t-tests on log-transformed intensities, pre and post normalization, when each missing intensities in GSE or control gel were replaced by randomly selecting one of the 30 lowest spot intensity values from the 30 gels in the experiment. Spots in **bold **were later identified by MALDI-TOF. These were all spots that were biologically relevant to the system being studied.

**Table 9:** Comparing t-test results for the three different imputation methods

**Normalization 2 Missing replaced by = -17.28 Log transformed (100% of identified spots picked up)**	**Normalization 2 Missing replaced by Random sample of minimum from 15 gels in each group Log transformed (91% of identified spots picked up)**	**Normalization 2 Minimum replaced by Random sample of minimum from all 30 gels Log transformed (91% of identified spots picked up)**
**SSP 1134**	SSP1134	SSP 1134
**SSP 1509**	**SSP 1509**	**SSP 1509**
**SSP 1733**	**SSP 1733**	**SSP 1733**
**SSP 2309**	**SSP 2309**	**SSP 2309**
**SSP 3219**		
**SSP 3806**	**SSP 3806**	**SSP 3806**
**SSP 4203**	**SSP 4203**	**SSP 4203**
**SSP 4225**	**SSP 4225**	**SSP 4225**
**SSP 4435**	**SSP 4435**	**SSP 4435**
**SSP 4724**	SSP 4724	SSP 4724
**SSP 5413**		**SSP 5413**
**SSP 6205**	**SSP 6205**	**SSP 6205**
**SSP 6304**	SSP 6228	SSP 6228
**SSP 6314**	SSP 6304	SSP 6304
	SSP 6314	SSP 6314
**SSP 6452**	**SSP 6452**	**SSP 6452**
**SSP 7223**	SSP 7223	SSP 7223
	SSP 7334	SSP 7334
**SSP 7750**	SSP 7750	SSP 7750

In column 1 missing values were replaced with the lowest intensity value in experiment; in column 2 values to replace missing intensities were randomly chosen from the 15 lowest intensity values within a treatment group; in values to replace missing intensities were randomly chosen from the 30 lowest intensity values without regard to treatment group.

**Table 10:** Comparing variances of spot intensities when using multiple values to impute versus a single value imputation.

	**Multiple Imputation**		**Single Imputation**		
	**Treatment**	**Control**	**Treatment**	**Control**	

**SSP 1509**	0.767	0.663	7.05	8.39	**9 missing control**
	Range = (3.16)	Range = (2.63)	Range = (7.34)	Range = (6.56)	**3 missing treatment**
**SSP 1733**	0.356	1.4249	0.356	1.4249	**0 missing control**
	Range = (2.23)	Range = (3.74)	Range = (2.23)	Range = (3.74)	**0 missing treatment**
**SSP 6314**	1.2055	1.178	3.41	1.17	**0 missing control**
	Range = (3.94)	Range = (3.51)	Range = (7.15)	Range = (3.51)	**14 missing treatment**
**SSP 6452**	0.547	0.564	0.547	0	**15 missing control**
	Range = (2.79)	Range = (2.14)	Range = (2.79)	Range = (0)	**0 missing in treatment **

The variances for spots with missing values are either under estimated or over estimated with single imputation values. The ranges and variance values of intensities are closer to that of SSP 1733 (spot with no missing intensities) in the case of multiple value imputation.

**Table 11:** Comparing averaging across replicates versus not.

**Averaged across replicates (54% of identified spots picked up)**	**Log Normalized Log Transformed Missing replaced with – 17.28 (100% of identified spots picked up)**
SSP 1134	SSP 1134
**SSP 1509**	**SSP 1509**
**SSP 1733**	**SSP 1733**
	**SSP 2309**
**SSP 2864**	
SSP 3222	
	**SSP 3219**
	**SSP 3806**
	**SSP 4203**
**SSP 4225**	**SSP 4225**
	**SSP 4435**
	SSP 4724
**SSP 5413**	**SSP 5413**
	**SSP 6205**
SSP 6236	
	SSP 6304
SSP 6314	SSP 6314
SSP 6349	
**SSP 6452**	**SSP 6452**
SSP 7144	
	SSP 7223
SSP 7439	
SSP 7750	SSP 7750

Column 1 has the results of two sample t-tests when the intensity values were averaged across replicates. Columns 2 represents results of two sample t-tests when replicates were treated as independent observations.
